# Stress-induced lipocalin 2 remodels the tumor microenvironment to exclude T cells

**DOI:** 10.1186/s43556-026-00582-6

**Published:** 2026-09-19

**Authors:** Xinxiao Zhu, Jian Zhou, Fangfang Zhou

**Affiliations:** Departent of General Sugery, the First Affiliated Hospital of Soochow University, Institutes of Biology and Medical Sciences, Suzhou Medical College, Soochow University, Suzhou, 215006 China

In a recent study published in *Nature*, Bossowski and colleagues uncover that the integrated stress response (ISR) transcription factor ATF4 drives immune evasion through a cell-extrinsic mechanism: it induces the secreted protein lipocalin 2 (LCN2), which reprograms macrophages to suppress the T-cell chemoattractant CXCL9 and induce IL-6, thereby excluding CD8⁺ and CD4⁺ T cells from tumors [1]. These findings not only reveal an unexpected bridge between tumor stress adaptation and immune escape but also suggest that targeting LCN2, either alone or in combination with anti-PD-1 blockade, could serve as a promising strategy to enhance cancer immunotherapy.

Tumor cells navigate a hostile microenvironment characterized by hypoxia, nutrient deprivation, and metabolic waste accumulation [2, 3]. These conditions collectively pose significant stresses that must be overcome for survival and malignant progression. The integrated stress response (ISR) serves as a central adaptive mechanism in this context. It converges on the transcription factor ATF4 (Activating Transcription Factor 4) to orchestrate transcriptional programs that support metabolic adaptation, redox balance, and cell survival under stressful conditions [4]. However, while ATF4 is well established as a cell-intrinsic regulator of tumor fitness, its potential role in modulating anti-tumor immunity—whether through cell-extrinsic mechanisms—has remained largely unexplored. This gap is particularly striking given that many of the stresses that activate the ISR (hypoxia, nutrient scarcity) also profoundly shape the tumor immune microenvironment. Understanding whether and how the ISR-ATF4 axis bridges tumor stress adaptation with immune evasion could reveal new vulnerabilities for immunotherapy.

To interrogate the immune-dependent function of ATF4, the authors utilized CRISPR to delete *Atf4* in lung adenocarcinoma cells. Loss of *Atf4* dramatically impaired tumor growth in immunocompetent mice but had no effect in immunodeficient hosts, establishing that ATF4 promotes tumor progression primarily by suppressing anti-tumor immunity rather than by regulating cell-intrinsic proliferation or survival.

With this in hand, the authors performed an in vivo CRISPR screen targeting 477 ATF4-regulated genes and identified *Lcn2* as the top candidate. *Lcn2* knockout phenocopied *Atf4* loss—impaired growth in immunocompetent but not immunodeficient mice—and ectopic *Lcn2* expression fully rescued *Atf4*-deficient tumors, establishing LCN2 as the critical downstream effector. Notably, LCN2's immunosuppressive function required its secretion but was independent of its canonical iron-binding capacity, distinguishing its role in cancer from its antibacterial function. Given that ATF4 is the master transcriptional effector of the ISR, pharmacological inhibition of this pathway using ISRIB phenocopied the effects of *Atf4* and *Lcn2* loss, reinforcing the functional hierarchy from ISR activation to LCN2 secretion.

To elucidate how tumor-derived LCN2 sculpts the immune microenvironment, the authors turned to single-cell transcriptomics. Given that SLC22A17 had been previously characterized as a receptor for LCN2, they examined its expression in the single-cell dataset and found it was largely restricted to macrophages. This prompted the authors to investigate whether LCN2 signals through this receptor on macrophages to mediate immune suppression. Flow cytometry and functional assays subsequently confirmed that LCN2 signals through SLC22A17 on macrophages, suppressing the T cell chemoattractant Cxcl9 while inducing Il6, however, the precise intracellular signaling events that connect LCN2–SLC22A17 engagement to these transcriptional changes remain unknown. Future investigations are warranted to determine whether this pathway operates through canonical transcription factors such as NF-κB or STAT3, or involves epigenetic remodeling within tumor-associated macrophages, thereby driving macrophages toward an immunosuppressive interstitial state that impairs CD8⁺ and CD4⁺ T cell infiltration. Three-dimensional co-culture experiments further demonstrated that this LCN2-mediated T cell exclusion was macrophage-dependent and reversible by CXCL9 neutralization, pinpointing CXCL9 suppression as a critical node in the immune evasion pathway.

Having dissected the underlying mechanism, the authors next evaluated the clinical relevance of their findings. In tissue microarrays of lung and pancreatic adenocarcinoma, as well as in TCGA pan-cancer analyses, *LCN2* expression inversely correlated with T cell infiltration, positively correlated with tumor grade, and was associated with an “immune-excluded” tumor phenotype and poorer overall survival. Consistently, a retrospective analysis of 976 patients who received immunotherapy across multiple public datasets revealed that low *LCN2* expression correlated with improved clinical outcomes, suggesting its potential as a predictive biomarker for immunotherapy response.

From a therapeutic perspective, neutralizing antibodies against mouse and human LCN2 significantly suppressed tumor growth, extended survival, and increased T cell infiltration in orthotopic models of lung and pancreatic cancer. Using a "humanized" system (*Lcn2*⁻/⁻ KP cells expressing human LCN2 combined with anti-human LCN2 antibody), the authors demonstrated that targeting tumor-derived LCN2 alone is sufficient for therapeutic efficacy. Notably, combining anti-LCN2 with anti-PD-1 therapy further extended survival, highlighting the potential to overcome resistance to immune checkpoint blockade.

While these therapeutic results are encouraging, it is important to recognize that LCN2 is not merely a tumor-promoting factor but also a component of the innate immune system involved in host defense against bacterial infections and the maintenance of iron homeostasis. Systemic neutralization of LCN2 could therefore carry unintended risks, including off-target toxicities in non-tumor tissues and potential impairment of patients' normal immune functions. These concerns underscore the need for future studies to rigorously evaluate the safety profile of systemic LCN2 blockade and to explore tissue-specific or tumor-localized targeting strategies that might mitigate these risks while preserving therapeutic efficacy.

In conclusion, this study adds a new dimension to the understanding of ATF4's functional repertoire (Fig. 1). Alongside its recently discovered roles in chemotherapy-induced metastasis [4] and endothelial cell metabolism [5], the work positions ATF4 as a multifaceted regulator that integrates stress adaptation with immune modulation. Looking ahead, several questions merit further exploration. A major limitation of the current study is the lack of mechanistic detail downstream of SLC22A17. How LCN2 binding to this receptor leads to suppression of CXCL9 and induction of IL-6 remains unknown. The downstream signaling pathways (whether NF-κB, STAT3, or other transcription factors are involved) have not been delineated. Whether LCN2 directly regulates transcription of these genes or acts through post-transcriptional mechanisms also requires investigation. Addressing these gaps will be essential for therapeutic targeting. Beyond this, the context-dependent functions of LCN2 across different tumor types and immune landscapes warrant further study. From a therapeutic standpoint, the striking synergy between anti-LCN2 and anti-PD-1 therapy raises the possibility of overcoming immune checkpoint resistance, though careful evaluation of on-target off-tumor effects will be essential given LCN2's established roles in host defense. Collectively, this study provides a compelling foundation, but future work must fill the mechanistic gaps to fully exploit LCN2 as a therapeutic target.

**Fig. 1:**
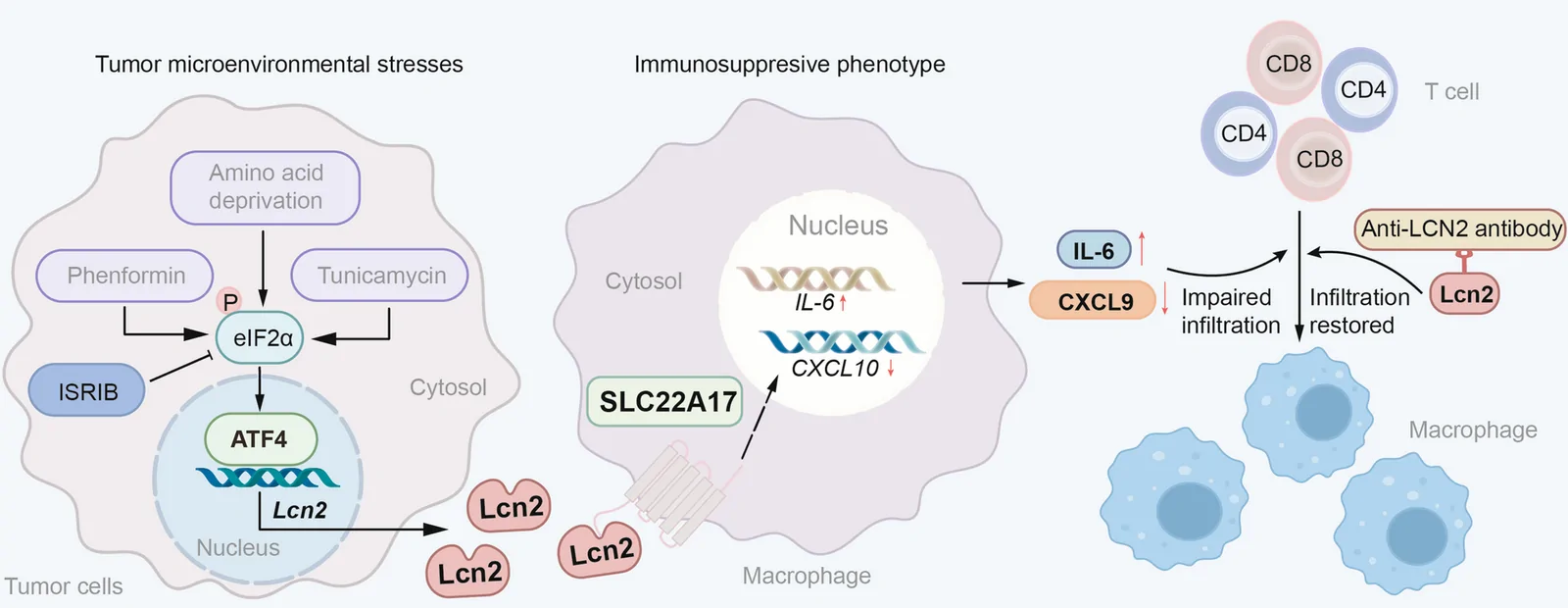
The ISR-ATF4-LCN2 axis promotes immune evasion by reprogramming tumor-associated macrophages. Tumor cells encounter microenvironmental stresses including hypoxia, nutrient deprivation, and metabolic waste accumulation. These stressors activate the integrated stress response (ISR), which converges on the transcription factor ATF4 to drive the expression and secretion of LCN2, a glycoprotein that is released into the tumor microenvironment. ISRIB, an allosteric inhibitor that disrupts p-eIF2α dimerization and consequently blocks ATF4 translation, suppresses LCN2 expression and phenocopies the effects of ATF4 loss in vivo. Secreted LCN2 binds to the SLC22A17 receptor on the surface of macrophages, triggering downstream signaling pathways that alter the macrophage transcriptional program—specifically suppressing the T-cell chemoattractant CXCL9 while inducing the pro-inflammatory cytokine IL-6. These changes drive macrophages toward an immunosuppressive interstitial phenotype. Consequently, CD8⁺ and CD4⁺ T cells are excluded from the tumor microenvironment, allowing tumor progression and immune evasion. Abbreviation: ISR, Integrated stress response; Eif2α, Eukaryotic Translation Initiation Factor 2 Alpha; ATF4, Activating Transcription Factor 4; ISRIB, Integrated Stress Response Inhibitor-B; LCN2, lipocalin 2; SLC22A17, Solute Carrier Family 22 Member 17; CXCL9, C-X-C Motif Chemokine Ligand 9; IL-6, Interleukin-6

## Data Availability

Not applicable.
